# Effect of non-surgical periodontal therapy on salivary pH, flow rate, salivary protein and calcium concentration in patients with chronic generalized periodontitis

**DOI:** 10.6026/973206300222885

**Published:** 2026-05-31

**Authors:** Anuja N Moharir, Anuja Hakkepatil, Vaishali Pagare, Prakash Gaikwad, Sabina Shaikh, Hiroj Bagde

**Affiliations:** 1 Department of Periodontology, D.Y. Patil Dental School, Lohegaon, Pune, Maharashtra, India; 2 Department of Conservative Dentistry, Endodontics and Aesthetic dentistry, DY Patil Dental School, Lohegaon, Pune, Maharashtra, India; 3 Department of Oral and Maxillofacial Surgery, DY Patil Dental School, Lohegaon, Pune, Maharashtra, India; 4 Department of Oral Pathology, Sinhagad Dental College and Hospital, Pune, Maharashtra, India; 5 Department of Periodontology, Government Dental College and Hospital, Chhatrapati Sambhajinagar, Maharashtra, India

**Keywords:** Saliva, salivary pH, salivary flow rate, non-surgical therapy

## Abstract

Periodontitis is an inflammatory disease of the supporting tissues of the tooth. The host-microbial interaction in periodontitis leads to ulceration of sulcular epithelium which acts as a portal of entry for the bacteria to enter the connective tissue and thus into the systemic circulation. Elimination of causative agent through non-surgical periodontal therapy causes a reduction in inflammation and periodontal microbes. Therefore, it is of interest to examine the effect of scaling on salivary flow rate, pH, salivary protein and calcium concentration. A total 50 patients were selected and they were assessed at their first visit and on 2nd day after non-surgical therapy. Unstimulated whole saliva was collected from patients at their first visit and flow rate was noted during collection of the sample. Salivary protein and calcium concentration, pH were estimated before and two days after non-surgical therapy. A positive correlation between the level of calcium and protein level reduction after therapy was seen.

## Background:

Periodontal disease is a multifactorial disease affected by both genetic and environmental factors. Salivary pH plays vital role in oral health because pH is a crucial factor in oral homeostasis [1]. As this is a chronic disease of the oral cavity comprises a group of inflammatory conditions affecting the supporting structures of the dentition [2, 3]. Dental plaque, a polymicrobial biofilm established on tooth surface is considered to be primary etiological factor [4]. Qualitative differences in dental plaque act as a factor of variability in an individual's risk for periodontal disease. Elimination of this factor through non-surgical periodontal therapy (scaling and root planing) causes a reduction in inflammation and periodontal microbes. Oral cavity is protected by numerous defensive functions of saliva which plays an important role. Saliva is the primary source for mineralization of supragingival plaque [5]. Rate of mineralization of saliva varies among individuals. As a mirror of oral and systemic health, saliva is a valuable source for clinically relevant information because it contains biomarkers specific for the unique physiologic aspects of periodontal diseases [6]. As an oral fluid it contains an abundance of proteins and genetic molecules and is readily accessible via totally noninvasive approach [7]. The changes of salivary proteins not only can reflect the physiologic and pathologic states but also subjected to oral and systemic regulation [8]. Proteins, especially albumin, are considered as a serum ultrafiltrate to the mouth. A major function of albumin is to provide colloid osmotic pressure in the plasma which prevents plasma loss from capillaries. Salivary protein and albumin concentrations were determined as markers for plasma protein leakage, occurring as a consequence of the inflammatory process. Onset of periodontitis is influenced by salivary calcium due to its affinity for being readily taken up by plaque [9]. High salivary calcium content would result in a more rapid rate of plaque mineralization and such individuals would be at increased risk for periodontal disease [10]. Therefore, it is of interest to evaluate the effect of non-surgical periodontal therapy on salivary flow rate, pH, salivary protein and calcium concentration in patients with chronic generalized periodontitis.

## Materials and Methods:

Fifty patients with chronic periodontitis coming to Department of Periodontology in D.Y. Patil Dental School, Pune were selected for the study. Written informed consent was obtained from each subject after explaining methodology of study. All patients underwent an intraoral examination. The criterion for gingivitis was based on criteria by National institute of dental research (NIDR).

NIDR- Gingival inflammation index (Bleeding Index)

0= No bleeding

1= Bleeding after probe is placed in gingival sulcus up to 2mm and drawn along inner surface of gingivalsulcus.

Criteria of periodontitis were based on loss of attachment with pocket depth of ≥5mm in atleast 8 sites.

## Exclusion criteria:

[1] Pregnant/ Lactating women.

[2] Patient with systemic disorder.

[3] Those undergoing radiation therapies.

[4] Hyper/ Hyposalivation of any origin.

[5] Patient taking any medication or vitamins supplements.

## Saliva samples and analyses:

Approximately 5ml of human whole unstimulated saliva was collected by spitting method, with the patients sitting in upright position. Patient was refrained from oral intake and tooth brushing for 2 hours prior to saliva collection. The sample was collected between 10 AM and 12 PM owing to its relation to the circadian cycle. Flow rate was calculated as volume collected divided by time required for the collection. Salivary calcium levels were detected using Liquizyme calcium detection kit (Mfg by Beacon Diagnostics Pvt. Limited India). Albumin and Total protein analysis was carried out using Liquix Total Protein kit (Mfg by ERBA diagnostics, Germany). Digital pH meter was used to estimate salivary pH. The pH meter was calibrated every day. The electrode was dipped in HCl of 0.1N overnight. The pH mater was then calibrated using freshly prepared buffers of pH 7 and pH 4. The later was used for finer adjustment to the pH. Following this the electrode was kept dipped in double distilled water. Prior to dipping the electrode in the sample it was gently dried completely using fresh sterile filter paper. After analyzing the pH, electrode tip was again washed with distilled water. The liquids and chemicals were freshly prepared every day. Saliva was collected on day one, following which the patient underwent scaling, after two days of scaling once again the samples were collected and analyzed for any change.

## Results:

The results were statistically analyzed by paired t test. Table 1 to 4 compares the parameters pH, flow rate, calcium and protein levels before and after scaling procedure. Of all parameters there was increase in pH value after scaling was carried out. Regarding pH mean and standard deviations of measurement of variables before and after scaling were 7.39±0.87 and 7.78±0.88with mean difference of 0.39±0.01 which was statistically significant with p <0.001 (Table 1). Other parameters showed decrease in values after oral prophylaxis which was statistically significant, flow rate decreased from 0.48 ± 0.13 to 0.34±0.03 with mean difference of 0.14±0.10 (Table 2). Calcium and protein levels decreased from 4.54 ± 1.35 to 3.59 ± x0.98 with mean difference of 0.95±0.37 and from 1.29±0.62 to 0.97±0.38 with mean Difference of 0.32 ± 0.24 respectively (Table 3 and Table 4).

## Discussion:

Gingivitis and periodontitis are oral diseases that are characterized by chronic inflammation. Dental biofilm, also known as plaque, initially develops supragingivally with mainly aerobic bacteria and matures over a period of several weeks. Over time, the flora changes from predominantly gram-positive to gram-negative, from facultative aerobes to strictly anaerobic species, with more motile forms present. Periodontal microbes trigger inflammatory response and results in higher levels of salivary albumin and total protein. Fifty patients diagnosed with chronic periodontitis attending Department of Periodontics were included in study. According to the study design the clinical parameters such as salivary flow rate, pH, salivary protein and calcium concentration were evaluated at the first visit. Scaling and root planing was performed on same day and patient was recalled after 2 days for reevaluation. Salivary sample was taken and same parameters were checked after 2 days. One of the studies carried out by Koppolu *et al.* has shown salivary pH levels evidently become alkaline in patients with chronic periodontitis post scaling and root planning [11, 12]. Since several factors influence salivary secretion and composition, standardized collection is essential so as to study the real functioning of gland. Human whole saliva is said to have a protein content of about 0.5 to 3 mg/mL in which parotid saliva has a protein content of about 0.4 to 4 mg/mL and submandibular, sublingual saliva of about 0.6 to 1.5mg/mL. Initially before scaling the protein concentration in saliva of patients diagnosed with periodontitis was found to be 1.29±0.62 mg/mL which has decreased to 0.97±0.38 mg/mL after scaling was carried out. The reduction in protein concentration was statistically significant. Salivary levels of alpha 2-macroglobulin, C-reactive protein, cathepsin G and elastase levels are directly related to an individual's periodontal status [13]. There are studies which showed increase in albumin concentration during inflammation and periodontal breakdown in saliva and gingival crevicular fluid [14]. One systematic review has proven that biomarkers in saliva can be used to monitor an early stage of periodontitis and progression of disease following therapy [15]. Composition of saliva is affected by protein contribution of glandular saliva, crevicular fluid protein and salivary flow rate. Significant rise of glandular derived proteins, cystatin C and amylase in periodontitis subjects proved the glandular origin of these proteins [16]. A number of studies have examined the correlation between nonezymatic, nonimmunoglobulin proteins such as mucins, lactoferrin, histatin, cystatin in saliva and periodontal disease. Periodontitis patients had significantly more *P. gingivalis*, *P. intermedia* and *T. denticola*. *T. denticola* increased the levels of salivary albumin and total protein as the proteins existing in the periodontal pocket are potential energy sources for *T. denticola* [17]. A study by Hollman and Van Der Hoeven (1999) has suggested that *T. denticola* occurs in close association with strong proteolytic bacteria such as *P. gingivalis* and *T. forsythia* in the subgingival plaque [18]. Therefore decrease in protein concentration after oral prophylaxis may be attributed to decrease in inflammation and decrease in periodontal microbes. One of the main factors affecting salivary composition is the flow index which varies in accordance with the type, intensity and duration of the stimulus. Normal salivary flow for stimulated and unstimulated saliva is 1 to 3 ml/ min and 0.25 to.35 ml/min respectively. In our study salivary flow rate decreased from 0.48±0.13 to 0.34±0.03 which was statistically significant. As the salivary flow increases, the concentrations of total protein, sodium, calcium, chloride and bicarbonate as well as the pH increases to various levels, whereas the concentrations of inorganic phosphate and magnesium diminish [19]. Thus the decrease in flow rate may be due to decrease in protein and calcium concentrations after oral prophylaxis was carried out. Greater the salivary flow, greater the consistency and greater the cleaning and diluting capacities; therefore, if changes in health cause a reduction in salivary flow, there would be a drastic alteration in the level of oral cleaning [20]. Normal range of salivary pH is from 6.2 to 7.6 and varies in accordance with the salivary flow from 5.3 to 7.8. Various sources of hydrogen ions in oral fluids are through secretion by the salivary glands in the form of organic and inorganic acids, production by the oral microbiota or acquisition through foods. These ions influence the equilibrium of calcium phosphates in the enamel. The higher the concentration of hydrogen ions, the lower the pH and vice versa. Significant increase in pH value after oral prophylaxis was noted from 7.39±0.87 to 7.78±0.88. pH variation was also noticed in a study carried out by Baliga *et al.* who concluded that salivary pH in patients with chronic generalized gingivitis was more alkaline than that in patients with clinically healthy gingiva. In patients with chronic generalized periodontitis, the salivary pH was more acidic than the control group [21]. The rate of mineralization of dental plaque and calculus formation is known to vary among individuals. High salivary calcium content would result in a more rapid rate of plaque mineralization. Since rapidly hardening plaque is difficult to clean and such individuals would be at increased risk for periodontal disease. Some earlier studies have shown positive correlation exists between high salivary calcium content and periodontitis [22] but others have contradicted this. The normal concentration of calcium in unstimulated whole mixed saliva is 4-6mg/dL. Calcium levels decreased from 4.54±1.35 to 3.59±0.98 after oral prophylaxis which was statistically significant. Increasing salivary flow rate and its resultant increase in pH above neutral [23, 24] is associated with an increase in both salivary calcium content [25] and periodontitis associated microorganisms. However, as dietary and systemic factors also affect salivary calcium and variations in salivary pH and flow rate may not necessarily correlate with salivary calcium content which may be modulated by systemic factors like liver function [26]. In one study it was observed that patients with higher education level were more adherent to oral healthcare and had less severe forms of periodontal disease [27]. This can be checked as additional factor in future studies on larger scale.

## Conclusion:

The effect of scaling on salivary flow rate, pH, salivary protein and calcium concentration is shown. Further, a positive correlation between the level of calcium and protein level reduction after therapy was seen. Thus, salivary composition can be considered as useful tool to check periodontal health.

## Figures and Tables

**Table 1:** Comparison pH before and after treatment

**ClinicalParameters**	**Scaling**	**MEAN±SD**	**Mean difference**	**P Value**
Ph	Pre-scaling	7.39±0.87	0.39±0.01	0.001
	Post scaling	7.78±0.88		Significant

**Table 2:** Comparison between flow rate before and after treatment

**Clinical Parameters**	**Scaling**	**MEAN±SD**	**Mean difference**	**P Value**
FLOW RATE	Pre-scaling	0.48±0.13	0.14±0.10	0
	Post scaling	0.34±0.03		Significant

**Table 3:** Comparison between calcium levels before and after treatment

**Clinical Parameters**	**Scaling**	**MEAN±SD**	**Mean difference**	**P Value**
CALCIUM	Pre-scaling	4.54±1.35	0.95±0.37	0
	Post scaling	3.59±0.98		Significant

**Table 4:** Comparison between protein concentration before and after treatment

**Clinical Parameters**	**Scaling**	**MEAN±SD**	**Mean difference**	**P Value**
PROTEIN	Pre-scaling	1.29±0.62	0.32±0.24	0
	Post scaling	0.97±0.38		Significant
